# Supplementary material for: Genetic transformation of the dinoflagellate chloroplast
Source: eLife. 2019 Jul 18;8:e45292. doi: 10.7554/eLife.45292 (PMC6639071; doi:10.7554/eLife.45292)
Supplement: Supplementary file 1. [file elife-45292-supp1.docx]

**Supplementary Data**

**Primer sequences**

MC-pG-F gactcttagaacgactaggctttctg
MC-pG-R GCGTAATCATGGTCATAGCTGTTTC (Tm 55^o^C, 1 min extension time)

MC-pG-F-II gtatcctcttctctccttgc
MC-pG-R-II CTCTCCCATATGGTCGAC (Tm 50^o^C, 1.5 min extension time)

CAT-FSS ACCTTGCCACTCGTCAC

CAT-F  GATATTTCTCAGTGGCATCGTAAG
CAT-R  GCTCGTTAAGCATACGACCTAC (Tm 60^o^C ,1.5 min extension time)

CAT-F-Nest CATCGTAAGGAACATTTCGAG
CAT-R-Nest CGACCTACATGGAAACCATC (Tm 50^o^C, 1.5 min extension time)

Copy-F TGAGGCCAGCGTAAACCTTT

Copy-R CGTTCAACCACACTTTATACAG

**pAmpChl sequence**

1 ctaaattgta agcgttaata ttttgttaaa attcgcgtta aatttttgtt aaatcagctc

61 attttttaac caataggccg aaatcggcaa aatcccttat aaatcaaaag aatagaccga

121 gatagggttg agtggccgct acagggcgct cccattcgcc attcaggctg cgcaactgtt

181 gggaagggcg tttcggtgcg ggcctcttcg ctattacgcc agctggcgaa agggggatgt

241 gctgcaaggc gattaagttg ggtaacgcca gggttttccc agtcacgacg ttgtaaaacg

301 acggccagtg agcgcgacgt aatacgactc actatagggc gaattggcgg aaggccgtca

361 aggccacgtg tcttgtccgc ttaattaaga ttaagaacga ttagattacc ttccttatta

421 ttgggaacgt ttgaacgatg aattaaactc acagtaggcc cttcgggcgc ctttcgaacg

481 acgtattgac ctacctacct aagtagagat cgttgtggcg acgtatcaat acccgggtaa

541 acaggtacaa ctgttgtatg gttatcctgc aacacctctt agatgctaga atgagtccat

601 aaaggcttgg aatctccgct tgtagttatc tctggttagg taatagtgcc tttccttctt

661 tgagcctcct accgaaagtc aattcaacag cacagatgag tattattaag agctcaaaca

721 atgttgatca aagacgatcg aaggatacgg aagcgtagag aatagacaat cgggagacta

781 tcattagtcc tggacaaatg ttgtcagaca tgaggttgac caggtcattc ataggctctc

841 cggtcatttt gttccatctc taccccagta gagaaaaatc caggtcatat cataggagat

901 ggaactgaga gatcgagaga acgaagacag aaaggaggtg aaaggtagtt ggcatgttga

961 tctggcgagt cagaggcatc aaacaggaac gaaggtaata gaatgaatga taattgatga

1021 aacgacaatg aaagtcctct aaagaagagg caactaataa agatgaaaca tatctcgggg

1081 gtgcgcctaa gtcaggctaa gaaagcatca aacagataca accgctgggc aggtcaacaa

1141 gtgattaatc ctgatctaag gatgattacg acctgcggaa aggtggttac aatacctcat

1201 acctgtatga ctgatcggtg ctttaatcac accttgcaag ttgccaatta cattgagtag

1261 gcatctttaa tagcttgtag ggttcacacc gcagtctaaa ggtagaaagg tccaacaatc

1321 aggtcatgtg gcccagaaac cacgctggta acctgagacc ctacctccct tcggggatcc

1381 tatcaaatgg tctgtgtatt cagtccccgt cttacggagt gcttgtggcg gtggagtggc

1441 aggggtaggc aagtagtcga gtggtgagct gtaatggtgt atccgcgttt accttcctgg

1501 tacctcggtg cctcggtaag aagacgaact ctcgttagtg aggccagcgt aaaccttttt

1561 ccatatctat tatggaaaag aagatcaccg gttacacaac cgtagatatt tctcagtggc

1621 atcgtaagga acatttcgag gcattccagt ctgtagcaca gtgtacatat aaccagacag

1681 tacagcttga tatcaccgca ttccttaaga cagtaaagaa gaacaagcac aagttctacc

1741 cagcattcat ccatatcctt gcacgtctta tgaacgcaca tccagagttc cgtatggcaa

1801 tgaaggatgg tgagcttgtc atctgggatt ctgtacatcc atgttataca gtattccatg

1861 agcagacaga gacattctct tccctttggt ctgagtatca tgatgatttc cgtcagttcc

1921 ttcatatcta ctctcaggat gtagcatgtt atggtgagaa ccttgcatat ttcccaaagg

1981 gtttcatcga aaatatgttc ttcgtatctg caaacccttg ggtatctttc acatctttcg

2041 atcttaacgt cgcgaacatg gacaatttct tcgcaccagt attcaccatg ggtaagtatt

2101 atacacaggg tgataaggtc cttatgccac ttgcaatcca ggtacatcat gcagtatgtg

2161 atggtttcca tgtaggtcgt atgcttaacg agcttcagca gtattgtgac gagtggcaag

2221 gtggtgcata gaatcaaccc ctttatccct ctctttggga ctcttagaac gactaggctt

2281 tctgattaac gatgatgtaa tattattaag aacgatggct agtaataatg cacgaaggcg

2341 cgccggagca caagactggc ctcatgggcc ttccgctcac tgcccgcttt ccagtcggga

2401 aacctgtcgt gccagctgca ttaacatggt catagctgtt tccttgcgta ttgggcgctc

2461 tccgcttcct cgctcactga ctcgctgcgc tcggtcgttc gggtaaagcc tggggtgcct

2521 aatgagcaaa aggccagcaa aaggccagga accgtaaaaa ggccgcgttg ctggcgtttt

2581 tccataggct ccgcccccct gacgagcatc acaaaaatcg acgctcaagt cagaggtggc

2641 gaaacccgac aggactataa agataccagg cgtttccccc tggaagctcc ctcgtgcgct

2701 ctcctgttcc gaccctgccg cttaccggat acctgtccgc ctttctccct tcgggaagcg

2761 tggcgctttc tcatagctca cgctgtaggt atctcagttc ggtgtaggtc gttcgctcca

2821 agctgggctg tgtgcacgaa ccccccgttc agcccgaccg ctgcgcctta tccggtaact

2881 atcgtcttga gtccaacccg gtaagacacg acttatcgcc actggcagca gccactggta

2941 acaggattag cagagcgagg tatgtaggcg gtgctacaga gttcttgaag tggtggccta

3001 actacggcta cactagaaga acagtatttg gtatctgcgc tctgctgaag ccagttacct

3061 tcggaaaaag agttggtagc tcttgatccg gcaaacaaac caccgctggt agcggtggtt

3121 tttttgtttg caagcagcag attacgcgca gaaaaaaagg atctcaagaa gatcctttga

3181 tcttttctac ggggtctgac gctcagtgga acgaaaactc acgttaaggg attttggtca

3241 tgagattatc aaaaaggatc ttcacctaga tccttttaaa ttaaaaatga agttttaaat

3301 caatctaaag tatatatgag taaacttggt ctgacagtta ccaatgctta atcagtgagg

3361 cacctatctc agcgatctgt ctatttcgtt catccatagt tgcctgactc cccgtcgtgt

3421 agataactac gatacgggag ggcttaccat ctggccccag tgctgcaatg ataccgcgag

3481 aaccacgctc accggctcca gatttatcag caataaacca gccagccgga agggccgagc

3541 gcagaagtgg tcctgcaact ttatccgcct ccatccagtc tattaattgt tgccgggaag

3601 ctagagtaag tagttcgcca gttaatagtt tgcgcaacgt tgttgccatt gctacaggca

3661 tcgtggtgtc acgctcgtcg tttggtatgg cttcattcag ctccggttcc caacgatcaa

3721 ggcgagttac atgatccccc atgttgtgca aaaaagcggt tagctccttc ggtcctccga

3781 tcgttgtcag aagtaagttg gccgcagtgt tatcactcat ggttatggca gcactgcata

3841 attctcttac tgtcatgcca tccgtaagat gcttttctgt gactggtgag tactcaacca

3901 agtcattctg agaatagtgt atgcggcgac cgagttgctc ttgcccggcg tcaatacggg

3961 ataataccgc gccacatagc agaactttaa aagtgctcat cattggaaaa cgttcttcgg

4021 ggcgaaaact ctcaaggatc ttaccgctgt tgagatccag ttcgatgtaa cccactcgtg

4081 cacccaactg atcttcagca tcttttactt tcaccagcgt ttctgggtga gcaaaaacag

4141 gaaggcaaaa tgccgcaaaa aagggaataa gggcgacacg gaaatgttga atactcatac

4201 tcttcctttt tcaatattat tgaagcattt atcagggtta ttgtctcatg agcggataca

4261 tatttgaatg tatttagaaa aataaacaaa taggggttcc gcgcacattt ccccgaaaag

4321 tgccac

**pAmpPSBA**

gggcgaattg ggcccgacgt cgcatgctcc cggccgccat ggcggccgcg ggaattcgat 60

agattaagaa cgattagatt accttcctta ttattgggaa cgtttgaacg atgaattaaa 120

ctcacagtag gcccttcggg cgcctttcga acgacgtatt gacctaccta cctaagtaga 180

gatcgttgtg gcgacgtatc aatacccggg taaacaggta caactgttgt atggttatcc 240

tgcaacacct cttagatgct agaatgagtc cataaaggct tggaatctcc gcttgtagtt 300

atctctggtt aggtaatagt gcctttcctt ctttgagcct cctaccgaaa gtcaattcaa 360

cagcacagat gagtattatt aagagctcaa acaatgttga tcaaagacga tcgaaggata 420

cggaagcgta gagaatagac aatcgggaga ctatcattag tcctggacaa atgttgtcag 480

acatgaggtt gaccaggtca ttcataggct ctccggtcat tttgttccat ctctacccca 540

gtagagaaaa atccaggtca tatcatagga gatggaactg agagatcgag agaacgaaga 600

cagaaaggag gtgaaaggta gttggcatgt tgatctggcg agtcagaggc atcaaacagg 660

aacgaaggta atagaatgaa tgataattga tgaaacgaca atgaaagtcc tctaaagaag 720

aggcaactaa taaagatgaa acatatctcg ggggtgcgcc taagtcaggc taagaaagca 780

tcaaacagat acaaccgctg ggcaggtcaa caagtgatta atcctgatct aaggatgatt 840

acgacctgcg gaaaggtggt tacaatacct catacctgta tgactgatcg gtgctttaat 900

cacaccttgc aagttgccaa ttacattgag taggcatctt taatagcttg tagggttcac 960

accgcagtct aaaggtagaa aggtccaaca atcaggtcat gtggcccaga aaccacgctg 1020

gtaacctgag accctacctc ccttcgggga tcctatcaaa tggtctgtgt attcagtccc 1080

cgtcttacgg agtgcttgtg gcggtggagt ggcaggggta ggcaagtagt cgagtggtga 1140

gctgtaatgg tgtatccgcg tttaccttcc tggtacctcg gtgcctcggt aagaagacga 1200

actctcgtta gtgaggccag cgtaaacctt tttccatatc tattatgaca agccttattc 1260

gctctaactc ctggggttct ttcgttcaaa caatcacttc ttcctctaac cgtctttata 1320

tcggttggtt cggtctcctc gtcttcccac ttctctccct tgctaccgta gcttatatca 1380

ctgctttctt ccttgctcct gcagtcgata ttgatggtat ccgtgagcca gttgcaggtt 1440

cccttatcta tggtaacaac atcatctctg gtgctgttat tccttcttct aacgcaatcg 1500

gtgtccattt ctatcctctc tgggaatccc ttggtcttga tgaatggctc tacaacggtg 1560

gtacttatca gttcgttgtg ttccacttct tcctcggtgt ctgcggttgg atgggtcgtg 1620

aatgggaatt ctcctatcgt ctcggtatgc gtccatggat cttcgtagca ttctccgctc 1680

caattgcggc agcagctgca gtgttcatta tttacccaat cggtcagggt tccttctccg 1740

atggtatgcc acttggtatc cagggtactt tcaacttcat gcttgtcttc caagctgagc 1800

acaagatcct catgcatcct ttccacattc ttggtgtagc aggtgtcttc ggtggttctc 1860

tcttctccgc aatgcacggt tctcttgtat cctcttctct ccttgcagag actgctggtt 1920

ctgagtccct taacaacggt tacgtattcg gtcaagagga tgagacttat tccatctctg 1980

cagcacacgc atatttcggt cgtcttatct tccagtatgc tggcttcaac aactcccgtt 2040

ctcttcactt cttccttgca gcttggcctg taattggtat ctggttcacc tctcttggtg 2100

ttgctactat ggcattcaac cttaacggtt tcaacttcaa ccagtctatt cttgacgaat 2160

ctggtcacta cattaactct tgggctgata tcctcaaccg tgctgatctt ggtatcgagg 2220

taatgcacga gcgtaacgct cataacttcc ctcttgatct tgcatagaat caaccccttt 2280

atccctctct ttgggactct tagaacgact aggctttctg attaacgatg atgtaatatt 2340

attaagaacg atggctagta ataatgcacg aatcactagt gaattcgcgg ccgcctgcag 2400

gtcgaccata tgggagagct cccaacgcgt tggatgcata gcttgagtat tctatagtgt 2460

cacctaaata gcttggcgta atcatggtca tagctgtttc ctgtgtgaaa ttgttatccg 2520

ctcacaattc cacacaacat acgagccgga agcataaagt gtaaagcctg gggtgcctaa 2580

tgagtgagct aactcacatt aattgcgttg cgctcactgc ccgctttcca gtcgggaaac 2640

ctgtcgtgcc agctgcatta atgaatcggc caacgcgcgg ggagaggcgg tttgcgtatt 2700

gggcgctctt ccgcttcctc gctcactgac tcgctgcgct cggtcgttcg gctgcggcga 2760

gcggtatcag ctcactcaaa ggcggtaata cggttatcca cagaatcagg ggataacgca 2820

ggaaagaaca tgtgagcaaa aggccagcaa aaggccagga accgtaaaaa ggccgcgttg 2880

ctggcgtttt tccataggct ccgcccccct gacgagcatc acaaaaatcg acgctcaagt 2940

cagaggtggc gaaacccgac aggactataa agataccagg cgtttccccc tggaagctcc 3000

ctcgtgcgct ctcctgttcc gaccctgccg cttaccggat acctgtccgc ctttctccct 3060

tcgggaagcg tggcgctttc tcatagctca cgctgtaggt atctcagttc ggtgtaggtc 3120

gttcgctcca agctgggctg tgtgcacgaa ccccccgttc agcccgaccg ctgcgcctta 3180

tccggtaact atcgtcttga gtccaacccg gtaagacacg acttatcgcc actggcagca 3240

gccactggta acaggattag cagagcgagg tatgtaggcg gtgctacaga gttcttgaag 3300

tggtggccta actacggcta cactagaaga acagtatttg gtatctgcgc tctgctgaag 3360

ccagttacct tcggaaaaag agttggtagc tcttgatccg gcaaacaaac caccgctggt 3420

agcggtggtt tttttgtttg caagcagcag attacgcgca gaaaaaaagg atctcaagaa 3480

gatcctttga tcttttctac ggggtctgac gctcagtgga acgaaaactc acgttaaggg 3540

attttggtca tgagattatc aaaaaggatc ttcacctaga tccttttaaa ttaaaaatga 3600

agttttaaat caatctaaag tatatatgag taaacttggt ctgacagtta ccaatgctta 3660

atcagtgagg cacctatctc agcgatctgt ctatttcgtt catccatagt tgcctgactc 3720

cccgtcgtgt agataactac gatacgggag ggcttaccat ctggccccag tgctgcaatg 3780

ataccgcgag acccacgctc accggctcca gatttatcag caataaacca gccagccgga 3840

agggccgagc gcagaagtgg tcctgcaact ttatccgcct ccatccagtc tattaattgt 3900

tgccgggaag ctagagtaag tagttcgcca gttaatagtt tgcgcaacgt tgttgccatt 3960

gctacaggca tcgtggtgtc acgctcgtcg tttggtatgg cttcattcag ctccggttcc 4020

caacgatcaa ggcgagttac atgatccccc atgttgtgca aaaaagcggt tagctccttc 4080

ggtcctccga tcgttgtcag aagtaagttg gccgcagtgt tatcactcat ggttatggca 4140

gcactgcata attctcttac tgtcatgcca tccgtaagat gcttttctgt gactggtgag 4200

tactcaacca agtcattctg agaatagtgt atgcggcgac cgagttgctc ttgcccggcg 4260

tcaatacggg ataataccgc gccacatagc agaactttaa aagtgctcat cattggaaaa 4320

cgttcttcgg ggcgaaaact ctcaaggatc ttaccgctgt tgagatccag ttcgatgtaa 4380

cccactcgtg cacccaactg atcttcagca tcttttactt tcaccagcgt ttctgggtga 4440

gcaaaaacag gaaggcaaaa tgccgcaaaa aagggaataa gggcgacacg gaaatgttga 4500

atactcatac tcttcctttt tcaatattat tgaagcattt atcagggtta ttgtctcatg 4560

agcggataca tatttgaatg tatttagaaa aataaacaaa taggggttcc gcgcacattt 4620

ccccgaaaag tgccacctga tgcggtgtga aataccgcac agatgcgtaa ggagaaaata 4680

ccgcatcagg aaattgtaag cgttaatatt ttgttaaaat tcgcgttaaa tttttgttaa 4740

atcagctcat tttttaacca ataggccgaa atcggcaaaa tcccttataa atcaaaagaa 4800

tagaccgaga tagggttgag tgttgttcca gtttggaaca agagtccact attaaagaac 4860

gtggactcca acgtcaaagg gcgaaaaacc gtctatcagg gcgatggccc actacgtgaa 4920

ccatcaccct aatcaagttt tttggggtcg aggtgccgta aagcactaaa tcggaaccct 4980

aaagggagcc cccgatttag agcttgacgg ggaaagccgg cgaacgtggc gagaaaggaa 5040

gggaagaaag cgaaaggagc gggcgctagg gcgctggcaa gtgtagcggt cacgctgcgc 5100

gtaaccacca cacccgccgc gcttaatgcg ccgctacagg gcgcgtccat tcgccattca 5160

ggctgcgcaa ctgttgggaa gggcgatcgg tgcgggcctc ttcgctatta cgccagctgg 5220

cgaaaggggg atgtgctgca aggcgattaa gttgggtaac gccagggttt tcccagtcac 5280

gacgttgtaa aacgacggcc agtgaattgt aatacgactc actata 5326
